# Oral Bacterial Microbiomes in Association with Potential Prediabetes Using Different Criteria of Diagnosis

**DOI:** 10.3390/ijerph18147436

**Published:** 2021-07-12

**Authors:** Kornwipa Rungrueang, Suraphong Yuma, Chanita Tantipoj, Siribang-on Piboonniyom Khovidhunkit, Pornpoj Fuangtharnthip, Thitima Thuramonwong, Muneedej Suwattipong, Sirirak Supa-amornkul

**Affiliations:** 1 Residency Training Program, Department of Advanced General Dentistry, Faculty of Dentistry, Mahidol University, Bangkok 10400, Thailand; kornwipakwang@gmail.com; 2 Department of Physics, Faculty of Science, Mahidol University, Bangkok 10400, Thailand; suraphong.yum@mahidol.edu; 3 Department of Advanced General Dentistry, Faculty of Dentistry, Mahidol University, Bangkok 10400, Thailand; chanita.tat@mahidol.edu (C.T.); siribangon.pib@mahidol.edu (S.-o.P.K.); pornpoj.fun@mahidol.ac.th (P.F.); 4 Dental Hospital, Faculty of Dentistry, Mahidol University, Bangkok 10400, Thailand; thitima.thu@mahidol.ac.th (T.T.); muneedej.suw@mahidol.edu (M.S.); 5 Mahidol International Dental School, Faculty of Dentistry, Mahidol University, Bangkok 10400, Thailand; 6 Pornchai Matangkasombut Center for Microbial Genomic, Faculty of Science, Mahidol University, Bangkok 10400, Thailand

**Keywords:** prediabetes, oral microbiome, saliva, 16S rRNA, HbA1c, FPG

## Abstract

This study aimed to find a potential biomarker that can be used to diagnose prediabetic condition by comparing the salivary bacterial microbiomes between Thai dental patients with normoglycemia (NG) and those with potential prediabetes (PPG) conditions. Thirty-three subjects were randomly recruited. Demographic data were collected along with oral examination and unstimulated salivary collections. The salivary bacterial microbiomes were identified by high-throughput sequencing on the V3–V4 region of the bacterial 16S rRNA gene. Microbiomes in this study were composed of 12 phyla, 19 classes, 29 orders, 56 families, 81 genera, and 184 species. To check the validity of the selection criterion for prediabetes, we adopted two separate criteria to divide samples into PPG and NG groups using glycated hemoglobin A1c (HbA1c) or fasting plasma glucose (FPG) levels. Using the HbA1c level resulted in the significant reduction of *Alloprevotella*, *Neisseria*, *Rothia,* and *Streptococcus* abundances in PPG compared with those in NG (*p*-value < 0.05). On the other hand, the abundance of *Absconditabacteriales* was significantly reduced whereas *Leptotrichia, Stomatobaculum,* and *Ruminococcaceae* increased in the PPG group when the samples were classified by the FPG level (*p*-value < 0.05). It is implied that the group classifying criterion should be carefully concerned when investigating relative abundances between groups. However, regardless of the criteria, *Rothia* is significantly dominant in the NG groups, suggesting that *Rothia* might be a potential prediabetic biomarker. Due to the small sample size of this study, further investigation with a larger sample size is necessary to ensure that *Rothia* can be a potential biomarker for prediabetes in Thai people.

## 1. Introduction

The prevalence of type 2 diabetes mellitus (T2DM) is increasing globally [1,2]. In 2019, the prevalence of diabetes mellitus (DM) in adults aged 20–79 years old was 9.3% or 463 million people worldwide. It is predicted to rise to 10.2% or 578 million in 2030 [1]. Impaired glucose tolerance (IGT) and impaired fasting glucose (IFG) conditions signify a risk of future development of T2DM [3,4]. The incidence of T2DM progression five years after diagnosis of IGT and IFG was estimated to be 26% and 50%, respectively [3]. T2DM has complications and co-morbidities along with neuropathy, nephropathy, retinopathy, micro-macrovascular, and periodontal diseases [1,5,6]. Early hyperglycemia detection is a key to control and prevent T2DM [7].

The Liverpool declaration: promoting oral health in the 21st century stated that countries should provide supports to prevent and reduce oral and general diseases [8]. The statement emphasized the role of dentists in oral health promotion but also in general health promotion. Dental checkup or treatment provides an opportunity for dentists to meet their patients more regularly than the annual physical examination that is usually once a year. Tantipoj et al. (2018) reported that around 80 % of dental patients in Thailand are willing to have diabetes screening from saliva/oral fluid collection [9]. The prevalence of undiagnosed hyperglycemia in Thai dental patients was 33.8%, suggesting a high percentage of undiagnosed hyperglycemia in the Thai population [10].

The hyperglycemic diagnosis is currently based on fasting plasma glucose (FPG) and HbA1c levels [1]. These two methods have different pros and cons. For example, the HbA1c level can determine the chronic hyperglycemia, but FPG cannot. However, HbA1c strongly depends on ethnography, leading to different and complicated diagnosis criteria [11]. Using FPG requires fasting prior to the assessment. In addition, both levels are determined from a blood sample, which is invasive and unpleasant for some patients. An alternative approach for primary diagnosis of hyperglycemia is desirable. 

The glucose level in saliva and gingival crevicular fluid is found to be associated with hyperglycemic conditions [12,13,14]. Many studies reported that the salivary pH in adults and adolescents with diabetes is lower than in those with healthy condition [15,16,17]. Both glucose level and pH of saliva affect the oral bacterial community. The high level of saliva glucose could be a supplementary source of nutrition for certain bacterial species [18]. Acidification of saliva could interfere with bacterial reproduction, leading to an increase of *Firmicute* and decrease of *Bacteroidetes*, for instance [19]. Salivary acidification could also alter the oral microbiomes that develop dental caries, such as *Bifidobacterium dentium*, *Bifidobacterium longum*, and *S. mutans* [20]. Investigating the bacterial microbiome in saliva can be an alternative, noninvasive approach in diagnosing prediabetes and diabetes in the early state.

Recently, high-throughput sequencing of 16S rRNA genes methods have been developed to study the oral microbiome profile. This technique provides insight into the bacterial diversity and community structure of healthy people and those with diseases [21,22,23]. Using 16s rRNA sequencing, many studies reported a clear change in bacterial diversity in subgingival plaque, supra gingival plaque, and saliva of patients with T2DM [24,25,26]. In other words, obese individuals with T2DM showed a lower level of *Bifidobactera* than that of healthy participants [27], while *Leptotrichia, Staphylococcus, Catonella, and Bulleidia* are the genera whose abundances increased in the diabetes group [28]. To date, the studies of bacteria association have focused on the extreme cases of the disease condition, i.e., healthy or already having T2DM. The bacteria mentioned above might not be used as a biomarker to screen the prediabetes condition. Early diagnosis of diabetes with a noninvasive approach may provide a great chance to reduce the severity of the disease. It is thus important to examine the condition at the earliest state of potential prediabetes. Although many studies have already reported the association of relative abundances and the glycemic level in various countries [28,29], different demographics, genetics, and foods may affect the bacterial diversity. 

In this work, we study the diversity of the saliva bacterial microbiome in Thai dental patients using 16S rRNA amplification sequencing. We compare the resulting bacterial diversity found in healthy patients with that of patients who are potentially in the prediabetic state in an attempt to find a plausible biomarker that can be used to screen or diagnose diabetes in its early state.

## 2. Materials and Methods

### 2.1. Human Subjects

This study was conducted from May to December 2018 under the approval of the ethics committees of the Faculty of Dentistry/Faculty of Pharmacy, Mahidol University Bangkok, Thailand under the process number of MU-DT/PY-IRB 2017/047.2308. The primary criteria were that participants must be older than 32 years old, provide written informed consent, and be willing to comply with study procedures. It is found that the T2DM occurrence significantly increases at ages around 30–35 years old [30]. Thirty-four adult participants from the primary and emergency unit at the Faculty of Dentistry, Mahidol University, Bangkok, Thailand voluntarily participated in this study. We excluded participants who had one or more of the following conditions: diabetes, conditions that cause secondary diabetes (i.e., pancreatic cancer, Cushing’s syndrome, and acromegaly), pregnant, on steroid drugs or taking glucose-lowering medication or on chemotherapy, some systemic diseases (renal failure, hepatitis, immunodeficiency, severe anemia, and polycythemia), and taking antibiotic prophylaxis within 3 months and smoking within 5 years. Eventually, we had 33 participants who satisfied the selection criteria. The glycemic condition of all participants, including HbA1c and FPG, was examined by using hospital-based laboratory methods at the Faculty of Tropical Medicine, Mahidol University. The participants were classified into two groups, normoglycemia group (NG) and potential prediabetes group (PPG), by using the levels of either HbA1c or FPG independently. The participants were identified as a PPG if they had either the HbA1C level of 5.7–6.4% (PPG_HbA1c_) or the FPG level of 100–125 mg/dL (PPG_FPG_). The remaining participants were classified as normoglycemia; i.e., those with HbA1C less than 5.7% (NG_HbA1c_) or those with FPG less than 100 mg/dL (NG_FPG_) [31].

### 2.2. Data Collection

Participants needed to complete all three parts of the research procedure, consisting of demographic data collection and physical examination, oral examination, and saliva collection.

#### 2.2.1. Demographic Data and Physical Examination

The demographic data of the participants were collected by using a standardized questionnaire. This contained questions on age, gender, marital status, sugar consumption, frequency of sugar consumption, and family history of DM. Body weight and height of the participants were recorded in order to determine the body mass index (BMI), which was calculated as a weight in kilograms divided by the square of height in the unit of squared meters. Overweight was normally defined with BMI higher than 23 kg/m^2^ [32]. Systolic and diastolic blood pressures (SBP and DBP) were also recorded. Hypertension was defined as SBP and DBP higher than 140 mmHg and 90 mmHg, respectively [33].

#### 2.2.2. Oral Examination

The number of dental caries teeth (D), missing teeth (M), and filling teeth (F) were recorded and used to determine the decayed, missing, and filling teeth (DMFT) index. pH of saliva was determined by using pH indicator strips (Merck). Periodontal examination was performed following the process described by Tantipoj et al. (2017) [10]. Periodontal status was categorized into three levels: severe, moderate, and mild/no periodontitis, based on the criteria from the Centers for Disease Control (CDC) [34]. 

#### 2.2.3. Saliva Collection

The participants were instructed not to intake any food or beverage for at least 1 h prior to the oral examination and saliva collection. Then, they were asked to pool the saliva in the mouth for 4 min and perform passive drool into the 50 mL conical sterile polypropylene tube containing 2 mL of RNA *later*^®^ (Qiagen, Valencia, CA, USA). The saliva tube was then immediately stored at −35 °C until used.

### 2.3. DNA Extraction and 16s rDNA Sequencing

One milliliter of the saliva sample was thawed at room temperature and homogenized by vortex. QIAamp^®^ DNA Minikit (Qiagen, Valencia, CA, USA) was employed for DNA extraction. Extracted DNA samples were sent to Vishuo Biomedical in Singapore to perform 16S rDNA sequencing by Illumina Miseq. In short, a MetaVx™ Library Preparation kit (Genewiz, South Plainfield, NJ, USA) was used in library preparation. A V3–V4 region of rDNA was selected as a target. The amplicons were generated using forward primers containing the sequence 5′-CCTACGGRRBGCASCAGKVRVGAAT-3′ and reverse primers containing the sequence 5′-GGACTACNVGGGTWTCTAATCC-3′. DNA libraries were multiplexed and loaded on Illumina MiSeq according to the manufacturer’s instructions (Illumina, San Diego, CA, USA). Sequencing was performed using a 2 × 300 base pair. Raw 16s rRNA sequencing data are available with bioproject accession number PRJNA736207.

### 2.4. Sequencing Data Analysis

The sequencing analysis was performed with QIIME2 (version 2020.11) mainly based on protocols published by Estaki et al. (2020) [35]. Raw sequencing reads were processed using Cutadapt (version 1.9.1) to remove the primer sequences. The low-quality sequences were removed using the denoising method via the QIIME2 DADA2 (q2-dada2) plugin, which was found to be better than the traditional clustering method [35]. Taxonomy affiliations from phylum to genus levels were assigned to the remaining high-quality sequences by using the q2-feature-classifier plugin. The Naïve Bayes classifier was trained on the 16S rRNA reference sequences obtained from the SILVA release 132 rRNA database at 99% sequence similarity (https://www.arb-silva.de/ accessed on 20 November 2020) [36]. Alpha and beta diversity indices were determined by QIIME2. The potential biomarker of bacteria genera of each glycemic level was determined by linear discriminant analysis effect size (LefSe) and the genus with linear discriminate analysis (LDA). A score more than 2 was considered significantly different between group [37].

### 2.5. Statistical Analyses

Descriptive statistics were applied to examine the distributions of sociodemographic, medical history, clinical, and oral characteristics. Specifically, Fisher’s exact test was used to analyze the association of dichotomous and multiple categorial variables. The differences of continuous variables among the participants in NG and PPG groups, including the relative abundance of salivary microbiome, were examined by either the independent *t*-test or the Mann–Whitney U test depending on whether the distributions of those variables are normal or not. The statistical analyses were conducted by SPSS (SPSS statistical software, version 18.0 (IBM Corp., Armonk, NY, USA).

## 3. Results

### 3.1. Demographic and Oral Characteristics

As mentioned in Section 2.1, the final number of participants who satisfied all selection criteria was 33. Using the HbA1c level, we could classify 11 participants as PPG_HbA1c_, while the remaining 22 volunteers were classified as NG_HbA1c_. In contrast, only six participants were classified as PPG_FPG_ when applying the FPG level. It is noteworthy that the participants who were classified as PPG_FPG_ were not always PPG_HbA1c_ and vice versa. Two volunteers in PPG_FPG_ were from NG_HbA1c_, whereas seven PPG_HbA1c_ participants were classified as NG_FPG_. The demographic and physical characteristics of all participants are summarized in Table 1. The statistical comparison between NG and PPG samples was examined using the Fishers’ exact test. Regardless of the method used to classify the PPG sample, no significant difference of demographic and physical characteristics has been found between NG and PPG groups.

Oral characteristics of participants in NG and PPG groups were investigated and are listed in Table 2. The mean DMFT indices of both PPG groups (PPG_HbA1c_ and PPG_FPG_) seem to be higher than those of the NG groups, but they are actually not statistically different. Likewise, the distributions of saliva pH and periodontal status of the PPG sample are not significantly different from those of the NG group.

### 3.2. Global Sequencing Data

We acquired a set of 9,752,494 raw reads after sequencing the 16S rRNA V3–V4 hypervariable region from 33 DNA samples used in the study. The number of sequences per sample ranged from 202,498 to 390,570 reads with an average of 286,838 sequences per sample. After joining the paired-end sequences for each sample, the sequences underwent quality and size filtering. The final number of total sequences was 4,482,746. The number of sequences per sample ranged from 94,082 to 185,137 with an average of 131,845 sequences per sample. The average sequence length was 348 bps, with maximum length of 438 bps and the shortest length of 249 bps.

### 3.3. Bacterial Abundance and Distribution

**Figure 1:**
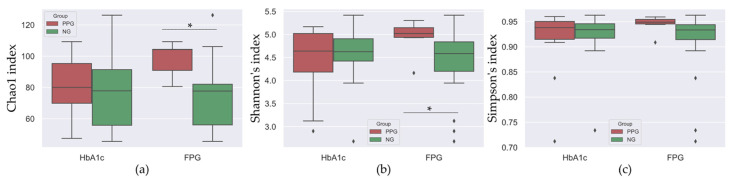
Chao1 (**a**), Shannon’s (**b**), and Simpson’s (**c**) diversity indices of saliva microbiomes in each sample group. In each panel, the PPG and NG groups divided by Hb1Ac and FPG levels are shown separately. The boxplots with red and green colors indicate 25% and 75% of the indices in PPG and NG groups, respectively. The diamond symbols are the outliers of the boxplot. The asterisks in (**a**,**b**) panels indicate the categories in which KS test reports have a significant difference (*p* < 0.05).

Alpha diversity indices were determined to investigate the diversity differences, if existing, between NG and PPG groups. Chao1 index was used to examine the species abundances, while Shannon’s and Simpson’s indices were used to evaluate diversity of oral microbiota in each group of samples (Figure 1). As seen in the left and middle panels of Figure 1, the PPG_FPG_ sample had the oral microbial abundance and diversity higher than those of the NG_FPG_ group. The independent *t*-test results show that the mean values of the abundance and diversity of the PPG_FPG_ are statistically different from those of NG_FPG_ with *p* = 0.001 (Chao1 index) and *p* = 0.03 (Shannon’s index), respectively. Using Kolmogorov–Smirnov (KS) test, we can reject the null hypothesis that the distributions of the Chao1 and Shannon’s indices of PPG_FPG_ are drawn from the same distribution as compared to those of the NG_FPG_ group at significance level of 0.005 and 0.009, respectively. In contrast, no significant difference of both abundance and diversity was found in case of using the HbA1c level to divide the PPG and NG groups. Mean and standard deviation of Chao1, Simpson’s, and Shannon’s indices are listed in Table S1.

**Figure 2:**
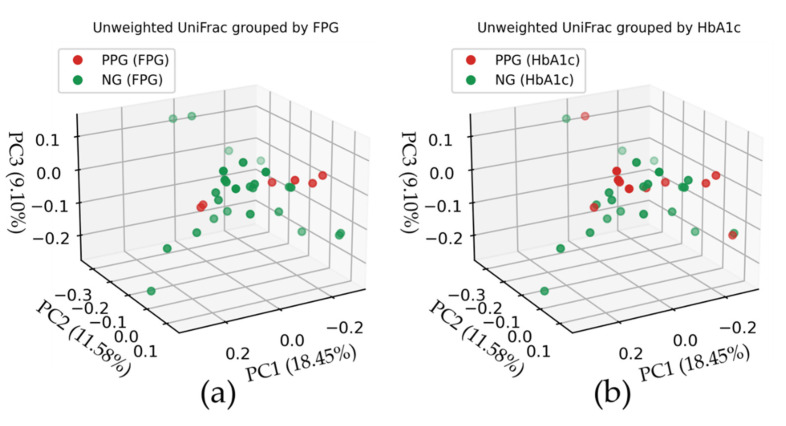

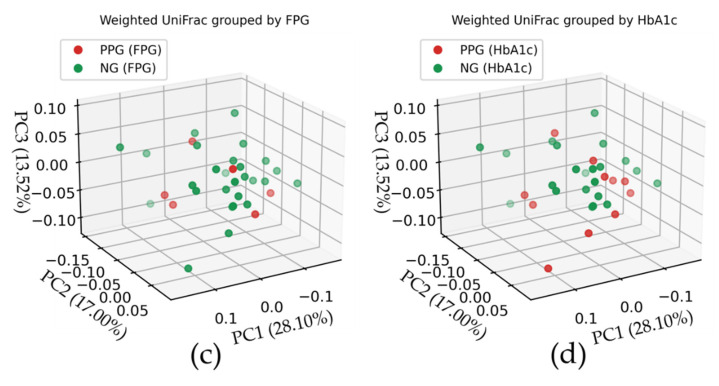
Principle coordinate analysis (PCoA) based on the unweighted (top panels) and weighted (bottom panels) UniFrac distance matrices. The red and green dots represent the PPG and NG groups, respectively. The colored symbols are shaded according to their positions on the 3D plot. The top panels (**a**,**b**) show the PCoA of unweighted UniFrac indices, while the bottom panels (**c**,**d**) show those of weighted UniFrac indices. The samples in the left panels (**a**,**c**) are PPG_FPG_ and NG_FPG_ groups, whereas those in the right panels (**b**,**d**) are based on the HbA1c level.

The principal component analysis (PCoA) was performed based on unweighted and weighted UniFrac distance matrices to evaluate the beta diversity between the PPG and NG groups. In unweighted PCoA analysis (top panels of Figure 2), the first principal coordinate (PC1) explained 18.45% of the total microbiome variations, while the second and third coordinates (PC2 and PC3) explained 11.58% and 9.10%, respectively. In weighted PCoA analysis (bottom panels of Figure 2), PC1, PC2, and PC3 explained 28.10%, 17.00%, and 13.52% of the total variations, respectively. No correlation or well-separated cluster is seen in all cases regardless of the criteria we used to classify the PPG and NG groups (Figure 2). It is suggested that the structures of bacterial community in PPG and NG groups were similar.

### 3.4. Bacterial Community Structure

**Figure 3:**
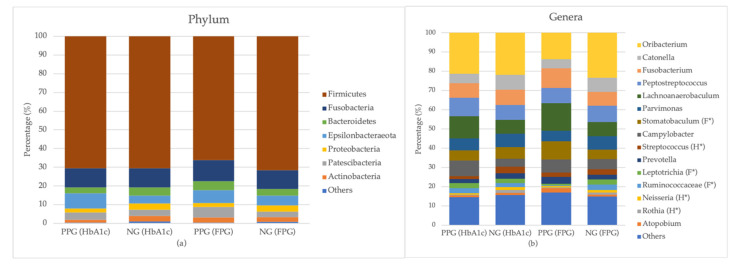
Bar plots of taxonomic profiles of patients in PPG and NG groups at the phylum level (**a**) and the genus level (**b**). Left to right bars in each panel shows the relative abundances of PPG_HbA1c_, NG_HbA1c_, PPG_FPG_, and NG_FPG_, respectively. Phyla and genera with the relative abundance below 1% are not directly shown in the figure, but included in others. H * and F * in parentheses of the right panel indicate the genus with significant difference (independent *t*-test with *p* < 0.05) between two groups (PPG and NG) in cases of using the HbA1c and FPG levels as criteria, respectively.

The bacterial distribution was characterized in terms of the relative taxonomic abundances. We found a total of 12 phyla, 19 classes, 29 orders, 56 families, 81 genera, and 184 species in the salivary samples. Ninety-nine percent of bacteria in all samples were from seven phyla. Firmicutes and Fusobacteria are the largest abundances of phyla in all samples, occupying roughly 80% of the total abundance (Figure 3a). The other five phyla with less abundance are Bacteroidetes, Epsilonbacteraeota, Proteobacteria, Patescibacteria, and Actinobacteria. In PPG_HbA1c_ and NG_HbA1c,_ Firmicutes was found at 70.68% ± 12.35% and 70.59% ± 11.05%, respectively. In PPG_FPG_ and NG_FPG_ groups, Firmicutes was found at 66.21% ± 10.48% and 71.60% ± 11.43%, respectively. We did not find any difference among the relative abundances at the phylum level between PPG and NG groups regardless of the group classifying criteria. Mean and standard deviation of all detected phyla are summarized in Table S2.

At the genus level, we found 81 genera of bacteria in total. In case of using the HbA1c level as a criterion, we found 56 common genera in PPG_HbA1c_ and NG_HbA1c_ groups, while 5 and 22 genera were identified only in PPG_HbA1c_ and NG_HbA1c_, respectively. In case of FPG, 48 genera were found in common, whereas 4 and 29 genera were found only in the PPG_FPG_ and NG_FPG_, respectively. The numbers of overlapping and unique genera found in all samples are illustrated in Figure S1. As seen in Figure 3b, the majority (80%) of bacteria were classified into 15 genera, of which average relative abundances and standard deviation are summarized in Table S3. The genus with the highest relative abundance in both PPG and NG groups was *Oribacterium* (PPG_HbA1c_ = 21.31% ± 16.56%, NG_HbA1c_ = 21.75% ± 17.34%, PPG_FPG_ = 13.73% ± 17.23%, and NG_FPG_ = 23.35% ± 16.5%). From the top 15 genera, *Rothia*, *Neisseria*, and *Streptococcus* in PPG_HbA1c_ were significantly lower than those in NG_HbA1c_ with *p*-values of *p =* 0.006, *p* = 0.036, and *p* = 0.001, respectively. In case of using the FPG level, PPG_FPG_ has lower abundances of *Ruminococcaceae* (*p* = 0.007) and *Leptotrichia* (*p =* 0.043) than NG_FPG_ does. Meanwhile, *Stomatobaculum* was found to show higher abundance in PPG_FPG_ as compared with NG_FPG_ (*p =* 0.034). Moreover, we also found two additional genera that are not in the top 15 genera but show significant differences in their relative abundances. *Alloprevotella* found in PPG_HbA1c_ has lower abundance than in NG_HbA1c_ (*p* = 0.043), whereas the relative abundance of *Absconditabacteriales* is found to be higher in PPG_FPG_ than in NG_FPG_ (*p =* 0.030). 

### 3.5. Differential Microbiota Compositions

**Figure 4:**
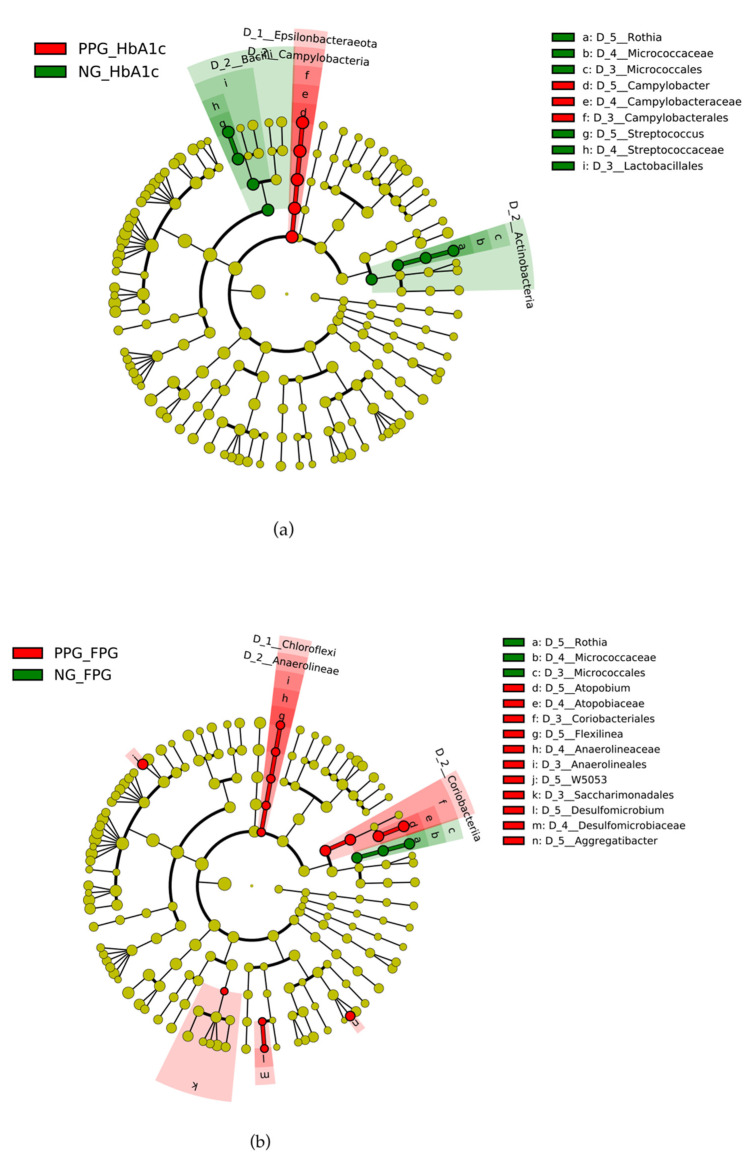
Cladograms from LEfSe analysis of saliva bacterial genera between PPG_HbA1c_ and NG_HbA1c_ (**a**) and between PPG_FPG_ and NG_FPG_ (**b**). The red and green labels show the genus of bacteria with significantly higher abundance in PPG and NG groups, respectively.

We examined possible biomarkers in the potential prediabetes sample using LEfSe analysis. The LEfSe results are illustrated by cladograms in Figure 4. Names of bacterial taxa appearing in the figure are those with significantly different abundances between prediabetes and normoglycemia groups. The *Rothia* genus in Actinobacteria phylum was enriched in both NG_HbA1c_ and NG_FPG_ groups as compared to their respective PPGs. Another genus with higher abundance in NG_HbA1c_ is *Streptococcus* in Firmicutes phylum (Figure 4a). On the other hand, *Campylobacter* in Epsilonbacteraeota phylum was more abundant in PPG_HbA1c_ than in NG_HbA1c_. Compared with NG_FPG_, PPG_FPG_ has more abundance in five genera, such as genus *Atopobium* in Actinobacteria and *Flexilinea* in Chloroflexi phylum (Figure 4b).

## 4. Discussion

We investigated the oral microbiome of patients with normal glycemic level and those who have a chance to develop hyperglycemia in the future. The ultimate goal of this study is to find a potential biomarker that we can use to diagnose the patients with high risk prior to the T2DM condition. The participants are divided into normoglycemia (NG) and potential prediabetes (PPG) groups based on their glucose level, determined by either HbA1c or FPG level. Regardless of the criteria used to divide the sample, the species richness of the PPG group was higher than that of NG. This increasing trend in microbial diversity is consistent with that found in T2DM patients [38,39,40]. In contrast, Saeb et al. (2019) found the opposite trend when comparing the impaired glucose tolerance and diabetes group with the normoglycemic group [29]. Chao1 and Shannon’s diversity indices of oral microbiome in PPG_FPG_ were higher than those in NG_FPG_, which is well in agreement with other studies of T2DM patients [40,41,42]. It is found that the oral microbiomes are potentially influenced by various factors such as oral health and host characteristics [43,44]. In this study, we found no significant difference of demographic, physical, and oral characteristics between the PPG and NG groups (Table 1 and Table 2). It is suggested that the differential abundances between PPG and NG groups found in this study are rarely due to demographics and characteristics of patients.

Firmicutes was the most abundant phylum found in both PPG and NG groups, which is similar to other studies [28,45]. It is indicated that Firmicute is the most abundant phylum in saliva and oral cavity microbiome [46]. Chen et al. (2020) found higher ratio of Firmicutes to Bacteroidetes (F/B ratio) in T2DM compared with healthy control [40]. The same trend of F/B ratio is found in our study as well, where the F/B ratio of PPG_HbA1c_ and NG_HbA1c_ was 22.55 and 16.83, respectively.

*Oribacterium* is a strictly anaerobic bacteria that can be used as a biomarker for saliva microbiome [46]. It was the most abundant genus in our samples. However, *Streptococcus* was the most abundant genus in the study in China [28], and *Prevoltella* was most abundant in patients in Saudi Arabia [29]. It is suggested that oral microbiome dysbiosis does not only depend on health condition, but also on host genetics, geography, diet, age, and habitat. *Streptococcus,* which is potentially associated with diabetes [28,40,42], was found within the top ten most abundant genera in our sample (Table S3). In fact, we even found higher abundance of *Streptococcus* in NG_HbA1c_ compared with that in PPG_HbA1c_. The result is consistent with Tantipoj et al. (2020) [47], who investigated the prevalence of *Streptococcus* in Thailand and found that *Streptococcus* abundance in normoglycemic group was marginally higher than that in hyperglycemic group. In contrast, Kampoo et al. (2014) showed that saliva *Streptococcus* was more abundant in diabetic patients as compared with the healthy participants from the southern part of Thailand [26]. Although all studies were carried out in Thailand, different methods have may affected the *Streptococcus* abundances. It is still skeptical to conclude whether or not *Streptococcus* might be a good biomarker for prediabetes.

**Figure 5:**
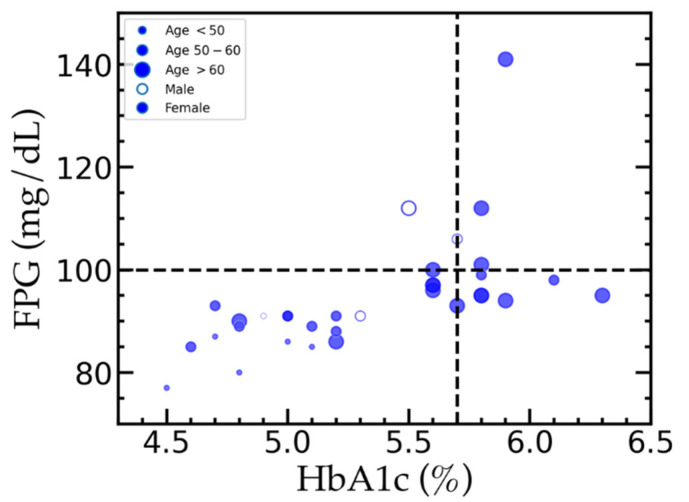
The distribution of blood sugar levels in studied population. The open and solid symbols represent male and female, respectively. Sizes of the symbols are related to ages of the participants. The vertical line at HbA1c of 5.7% indicates the HbA1c criterion used to divide PPG_HbA1c_ and NG_HbA1c_ groups. The horizontal line at FPG of 100 mg/dL is used to classify PPG_FPG_ and NG_FPG_ groups.

In the previous section, we found the differences in the relative abundances of some bacterial genera between PPG and NG groups. These differences changed when we used different criteria in classifying PPG and NG groups. For example, *Prevotella* was found more in NG_HbA1c_, while its abundance was lower in NG_FPG_ compared to the respective PPG groups (Table S3). Only *Rothia* showed the consistent trend of relative abundances between PPG and NG classified by both HbA1c and FPG criteria. Therefore, *Rothia* might be a potential prediabetic biomarker. As most of the resulting relative abundances between PPG and NG strongly depend on the group classifying criteria, we further investigated the samples by using both HbA1c and FPG levels as the criteria simultaneously to confirm if *Rothia* is really a potential prediabetic biomarker or not. Figure 5 shows the distributions of samples in the HbA1c–FPG space. The samples that were classified as PPG by using both criteria are located in the top right corner of the figure, while in the bottom left corner are those classified as NG. We found that 27.3 percent of participants were classified into different groups when using a different criterion (the top left and the bottom right corners of Figure 5). All of the participants younger than 50 years old were consistently classified into the same groups regardless of using HbA1c or FPG criteria. Guo et al. (2014) found that using only HbA1c to classify prediabetes increased the misdiagnosis rate in older population, while using both HbA1c and FPG can reduce the misdiagnosis rate [48]. This point needs to be considered when comparing the oral microbiome composition among different studies that may use different classifiers to separate prediabetes individuals from the normoglycemic group.

**Figure 6:**
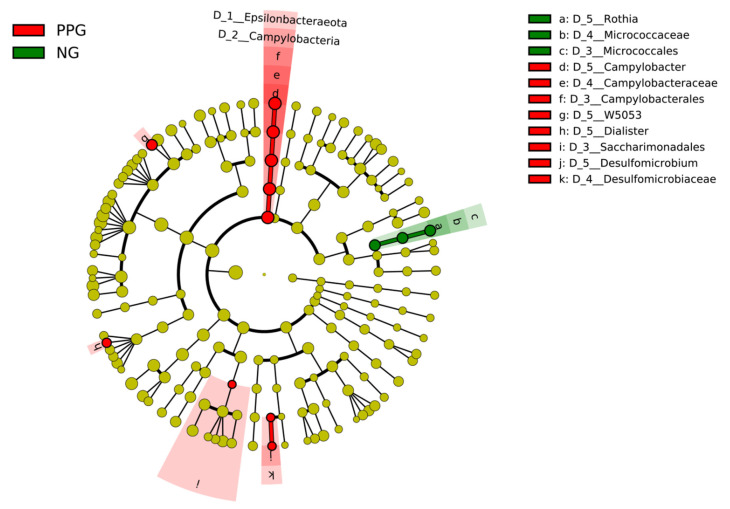
Cladogram from LEfSe analysis of saliva bacterial genera in PPG and NG groups classified by using both HbA1c and FPG levels. Green and red colors indicate the bacterial genera with higher abundance in PPG and NG, respectively.

We performed LefSe analysis of newly classified PPG and NG groups using both HbA1c and FPG levels (Figure 6). Unfortunately, only four participants were classified as PPG, whereas the rest were in normoglycemic group. *Rothia* still dominated in the NG group. *Rothia* is known as a nitrate-reducing oral bacterium, which plays an important role in balancing the oral cavity condition, such as producing alkali molecules to prevent acidity condition [49]. Caries-associated genera and periodontitis-associated genera were reported to reduce their growth under a nitrate condition [49]. Lundberg et al. (2018) also found antidiabetic effects after consuming dietary nitrate [50]. The nitrate reductase enzymes in the oral bacteria such as *Rothia* can metabolize nitrate into nitrite, which is further reduced into bioactive nitrogen oxides (NO) in blood and muscles [50]. NO synthesis is found to be an important mechanism to regulate the cardiovascular conditions and metabolisms, including an increase in the insulin secretion and glucose uptake of muscle [50]. As a result, a decrease in *Rothia* abundance can probably be a potential sign of having prediabetic condition.

As seen in Figure 6, *Campylobacter* was predicted to be another potential biomarker. We found higher abundance of *Campylobacter* in PPG samples compared with the NG group. *Campylobater rectus* shows a positive association with increasing blood glucose level [42]. Some species of *Campylobacter* were found more frequently in patients with periodontal disease compared with healthy control [51]. Periodontitis and diabetes condition can influence the severity of each other [6]. The increase of glucose level in PPG samples might thus promote growth of periodontitis-associated bacteria.

The limitation of this study is mainly the small sample size. The number of participants in each group after being classified into NG and PPG are not equally distributed. Although we have found potential biomarkers to diagnose the prediabetic condition, the larger sample size is still desirable to confirm the association.

## 5. Conclusions

We found that demographics and physical characteristics of the participants in the potential prediabetes group (PPG) and those in the normoglycemia group are similar regardless of the criterion (HbA1c or FPG). Meanwhile, the oral microbial abundance and diversity of PPG_FPG_ is statistically higher than those of NG_FPG_. The principal component analysis (PCoA) showed no correlation or well-separated cluster between both groups. According to LEfSe analysis, we found that using different criterion resulted in different bacterial abundances in groups. This should be considered when comparing the oral microbiomes among different studies. *Rothia* and *Campylobacter* are found to be the potential prediabetes-associated genera for elderly population in Thailand. Unfortunately, the sample size in this study was too small to declare a conclusive biomarker. Instead, *Rothia* and *Campylobacter* can be used as a guideline to select the potential prediabetic biomarker in future. Further studies with larger sample that can represent the entire Thai population are desirable.

## Figures and Tables

**Table 1:** Demographic and physical characteristics of 33 participants divided into NG and PPG groups according to either HbA1c or FPG levels.

Factor	Total *n* (%)	HbA1c			FPG		
		NG_HbA1c_ (<5.7%) *n* (%)	PPG_HbA1c_ (5.7–6.4%) *n* (%)	*p*-Value	NG_FPG_ (<100) *n* (%)	PPG_FPG_ (100–125) *n* (%)	*p*-Value
Age (year)				0.57			0.84
<50	6 (18.2)	3 (13.6)	3 (27.3)		5 (18.5)	1 (16.7)	
50–60	12 (36.4)	8 (36.4)	4 (36.4)		9 (33.3)	3 (50.0)	
>60	15 (45.5)	11 (50.0)	4 (36.4)		13 (48.2)	2 (33.3)	
Gender				0.28			
Male	4 (12.1)	4 (18.2)	0 (0.0)		4 (14.8)	0 (0.0)	1.00
Female	29 (87.9)	18 (81.8)	11 (100.0)		23 (85.2)	6 (100.0)	
Marital status				0.81			0.86
Single	13 (39.4)	8 (36.4)	5 (45.5)		10 (37.0)	3 (50.0)	
Married	11 (33.3)	7 (31.8)	4 (36.4)		9 (33.3)	2 (33.3)	
Divorced	9 (27.3)	7 (31.8)	2 (18.2)		8 (29.6)	1 (16.7)	
Sugar consumption				0.64			0.06
No	6 (18.2)	5 (22.7)	1 (9.1)		3 (11.1)	3 (50.0)	
Yes	27 (81.8)	17 (77.3)	10 (90.9)		24 (88.9)	3 (50.0)	
Frequency of Sugar consumption				1.00			1.00
<3 times/day	24 (72.7)	16 (72.7)	8 (72.7)		20 (74.1)	4 (66.7)	
≥3 times/day	9 (27.3)	6 (27.3)	3 (27.3)		7 (25.9)	2 (33.3)	
Family history of DM				0.71			1.00
No	18 (54.6)	11 (50.0)	7 (63.6)		15 (55.6)	3 (50.0)	
Yes	15 (45.5)	11 (50.0)	4 (36.4)		12 (44.4)	3 (50.0)	
Hypertension				0.37			1.00
No	27 (81.8)	19 (86.4)	8 (72.7)		22 (81.5)	5 (83.3)	
Yes	6 (18.2)	3 (13.6)	3 (27.3)		5 (18.5)	1 (16.7)	
Overweight				0.70			0.34
No	10 (30.3)	6 (27.3)	4 (36.4)		7 (25.9)	3 (50.0)	
Yes	23 (69.7)	16 (72.7)	7 (63.6)		20 (74.1)	3 (50.0)	
BMI (kg/m^2^)				0.67			1.00
Normal (<23)	14 (42.4)	12 (54.6)	2 (18.2)		11 (40.7)	3 (50.0)	
Obese (≥23)	19(57.6)	10 (45.5)	9 (81.8)		16 (59.3)	3 (50.0)	

Note: NG and PPG represent normoglycemia and potential prediabetes groups, respectively. FPG means the fasting plasma glucose level in the unit of mg/dL.

**Table 2:** Oral characteristics of 33 participants divided into NG and PPG groups according to either HbA1c or FPG levels.

Factor	Total *n* (%)	HbA1c			FPG		
		NG_HbA1c_ (<5.7%) *n* (%)	PPG_HbA1c_ (5.7–6.4%) *n* (%)	*p*-Value	NG_FPG_ (<100) *n* (%)	PPG_FPG_ (100–125) *n* (%)	*p*-Value
DMFT (mean)	13.45	12.05	16.27	0.08 ^t^	12.81	16.33	0.24 ^t^
pH of Saliva				0.84			0.29
Acid (<7)	23 (69.7)	16 (72.7)	7 (63.6)		17 (63.0)	6 (100.0)	
Neutral (=7)	6 (18.2)	4 (18.2)	2 (18.2)		6 (22.2)	0 (0.0)	
Base (>7)	4 (12.1)	2 (9.1)	2 (18.2)		4 (14.8)	0 (0.0)	
Periodontal status				0.38			0.83
No/mild	17 (51.5)	12 (54.6)	5 (45.5)		13 (48.2)	4 (66.7)	
Moderate	6 (18.2)	5 (22.7)	1 (9.1)		5 (18.5)	1 (16.7)	
Severe	10 (30.3)	5 (22.7)	5 (45.5)		9 (33.3)	1 (16.7)	

Note: NG and PPG represent normoglycemia and potential prediabetes groups, respectively. FPG means the fasting plasma glucose level in the unit of mg/dL. ^t^ indicates the use of the independent *t*-test.

## Data Availability

The data presented in this study are available on request from the corresponding author.

## Supplementary Materials

The following are available online at https://www.mdpi.com/article/10.3390/ijerph18147436/s1, Figure S1: Vann diagram of genera number in each study group; Table S1: Mean and standard deviation (SD) of each alpha diversity index in HbA1c and FPG groups, Table S2: Relative abundance of bacterial phyla in each glycemic classification group, Table S3: Relative abundance of bacterial genera in each glycemic classification group.
