# Supplementary material for: Oral Bacterial Microbiomes in Association with Potential Prediabetes Using Different Criteria of Diagnosis
Source: Int J Environ Res Public Health. 2021 Jul 12;18(14):7436. doi: 10.3390/ijerph18147436 (PMC8307246; doi:10.3390/ijerph18147436)
Supplement: Supplementary file 1 [file ijerph-18-07436-s001.zip › ijerph-1279800-supplementary.pdf]

Table S1. Mean and standard deviation (SD) of each alpha diversity index in HbA1c and FPG groups.

Index	HbA1c					FPG				
	NG		PPG		t-test	NG		PPG		t-test
	Mean	SD	Mean	SD	p-value	Mean	SD	Mean	SD	p-value
Simpson's	0.923	0.046	0.910	0.074	0.605	0.913	0.057	0.945	0.261	0.031
Shannon	4.557	0.566	4.442	0.786	0.672	4.4260	0.648	4.935	0.402	0.030
Choa1	76.395	21.603	81.047	20.209	0.549	73.444	0.060	98.202	11.671	0.002

NG mean normoglycemia group, PPG mean potential prediabetes group.

Table S2. Relative abundance of bacterial phyla in each glycemic classification group.

Phylum	HbA1c				FGP			
	NG (n=22)		PPG (n=11)		NG(n=27)		PPG (n=6)	
	Mean	Std. Deviation	Mean	Std. Deviation	Mean	Std. Deviation	Mean	Std. Deviation
Actinobacteria	2.92	3.378	1.42	1.525	2.34	3.157	2.81	1.962
Bacteroidetes	4.31	2.807	3.13	2.104	3.70	2.673	4.89	2.329
Chloroflexi	0.01	0.039	0.02	0.064	0.00	0.000	0.07	0.102
Cyanobacteria	0.25	1.173	0.00	0.000	0.20	1.058	0.00	0.000
Epsilonbacteraeota	4.25	7.525	8.07	4.836	5.23	7.253	6.85	5.470
Firmicutes	70.59	11.050	70.68	12.348	71.60	11.431	66.21	10.477
Fusobacteria	10.20	8.295	10.18	8.825	9.98	8.328	11.17	9.092
Patescibacteria	3.289	4.800	3.87	4.468	3.04	4.422	5.47	5.453
Proteobacteria	3.45	1.764	2.18	1.666	3.21	1.814	2.21	1.704
Spirochaetes	0.31	0.779	0.35	0.759	0.35	0.831	0.22	0.302
Synergistetes	0.36	1.356	0.09	0.221	0.30	1.226	0.12	0.294
Tenericutes	0.01	0.046	0.00	0.000	0.01	0.042	0.00	0.000
Bacteria	0.02	0.055	0.00	0.000	0.01	0.050	0.00	0.000
Unassigned	0.03	0.125	0.02	0.051	0.03	0.116	0.00	0.000

Table S3. Relative abundance of bacterial genera in each glycemic classification group.

Rank	Genus	HbA1c				FPG				LEfSe	
		NG (n=22)		PPG (n=11)		NG (n=27)		PPG (n=6)		HbA1c	FPG
		Mean	Std. Deviation	Mean	Std. Deviation	Mean	Std. Deviation	Mean	Std. Deviation		
1	Oribacterium	21.75	17.337	21.31	16.56	23.35	16.544	13.73	17.231		
2	Catonella	7.93	7.026	4.91	4.147	7.43	6.683	4.64	3.901		
3	Fusobacterium	7.85	7.913	7.55	5.832	7.19	6.769	10.26	9.144		
4	Peptostreptococcus	7.66	7.767	9.71	7.487	8.41	7.75	8.02	7.695		
5	Lachnoanaerobaculum	7.26	6.952	11.44	9.787	7.4	6.762	14.3	11.646		
6	Parvimonas	6.96	6.541	6.29	5.105	7.01	6.452	5.49	3.706		
7	Stomatobaculum (F*)	6.05	5.298	5.28	3.645	4.97	3.521	9.48	7.805		
8	Campylobacter	4.25	7.525	8.07	4.836	5.23	7.253	6.85	5.47	/	
9	Streptococcus(H*)	3.29	2.005	1.53	0.686	2.81	1.969	2.22	1.378	/	
10	Prevotella	2.87	2.021	2.10	1.47	2.42	1.891	3.51	1.605		
11	Leptotrichia(F*)	2.25	3.233	2.63	4.33	2.7	3.859	0.91	1.009		
12	Ruminococcaceae(F*)	2.17	3.057	2.71	5.215	2.8	4.101	0.34	0.828		
13	Neisseria(H*)	1.50	1.056	0.73	0.682	1.35	1.026	0.74	0.807		
14	Rothia(H*)	1.41	1.054	0.39	0.641	1.22	1.078	0.39	0.554	/	/
15	Atopobium	1.28	2.882	0.88	1.522	0.91	2.59	2.18	1.81		/
24	Alloprevotella (H*)	0.71	0.722	0.33	0.311	0.56	0.653	0.71	0.595		
26	Pasteurellaceae;_D5	0.56	0.738	0.64	0.933	0.68	0.847	0.17	0.255		/
37	D3_Clostridiales; D5_W5053	0.20	0.68	0.61	1.21	0.13	0.50	1.23	1.64		/
42	Absconditabacteriales (F*)	0.14	0.312	0.13	0.429	0.17	0.38	0	0		
43	Aggregatibacter	0.14	0.30	0.13	0.22	0.09	0.22	0.37	0.39		/
70	Flexilinea	0.01	0.04	0.02	0.06	0.00	0.00	0.07	0.10		/
80	Desulfomicrobium	0.00	0.00	0.01	0.04	0.00	0.00	0.02	0.05		/
-	Other	15.17	-	13.70	-	14.58	-	15.25	-		

Blue highlight indicated relative abundance of NG more than PPG.
Orange highlight indicated relative abundance of NG less than PPG.
H* indicate significantly different (p<0.05) between NG and PPG of HbA1c group using independent t-test.
F* indicate significantly different (p<0.05) between NG and PPG of FPG group using independent t-test.
/ indicate biomarker genera from LEfSe analysis.

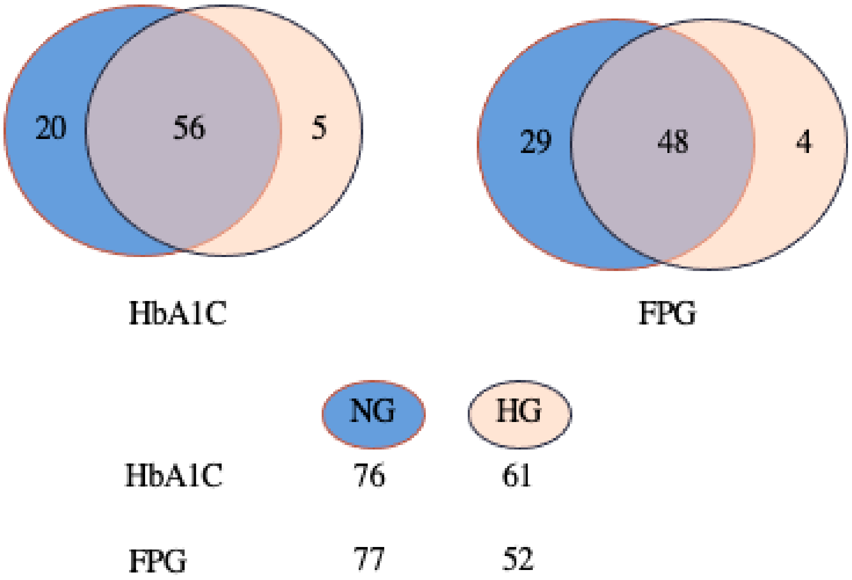

Figure S1. Vann diagram of genera number in each study group.
